# Supplementary material for: Conformational motions and ligand-binding underlying gating and regulation in IP3R channel
Source: Nat Commun. 2022 Nov 14;13:6942. doi: 10.1038/s41467-022-34574-1 (PMC9663519; doi:10.1038/s41467-022-34574-1)
Supplement: Supplementary file 1 — Supplementary Information [file 41467_2022_34574_MOESM1_ESM.pdf]

SUPPLEMENTARY FIGURES

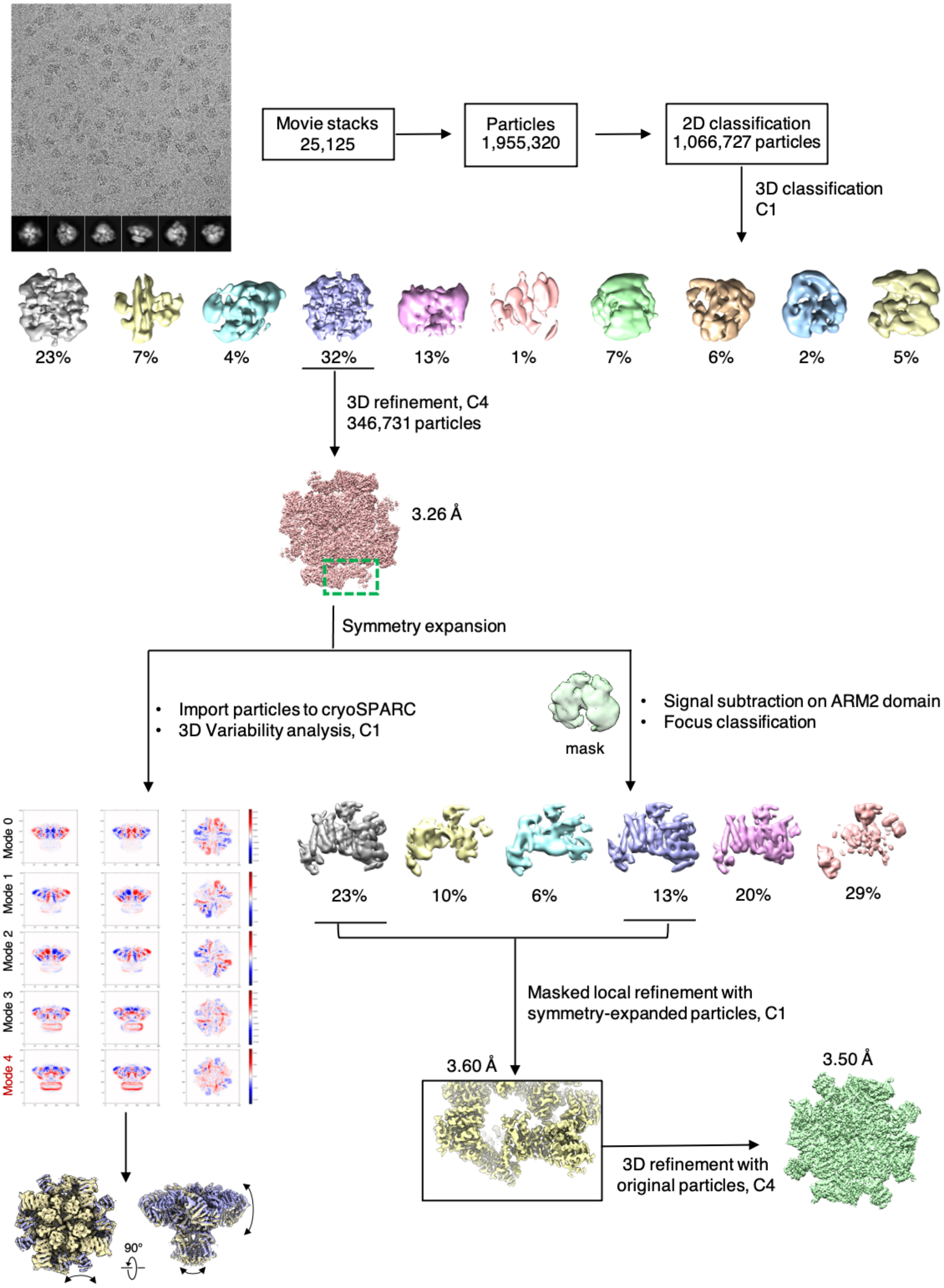

Supplementary Figure 1. Cryo-EM processing workflow for Ca-IP$_3$R1.

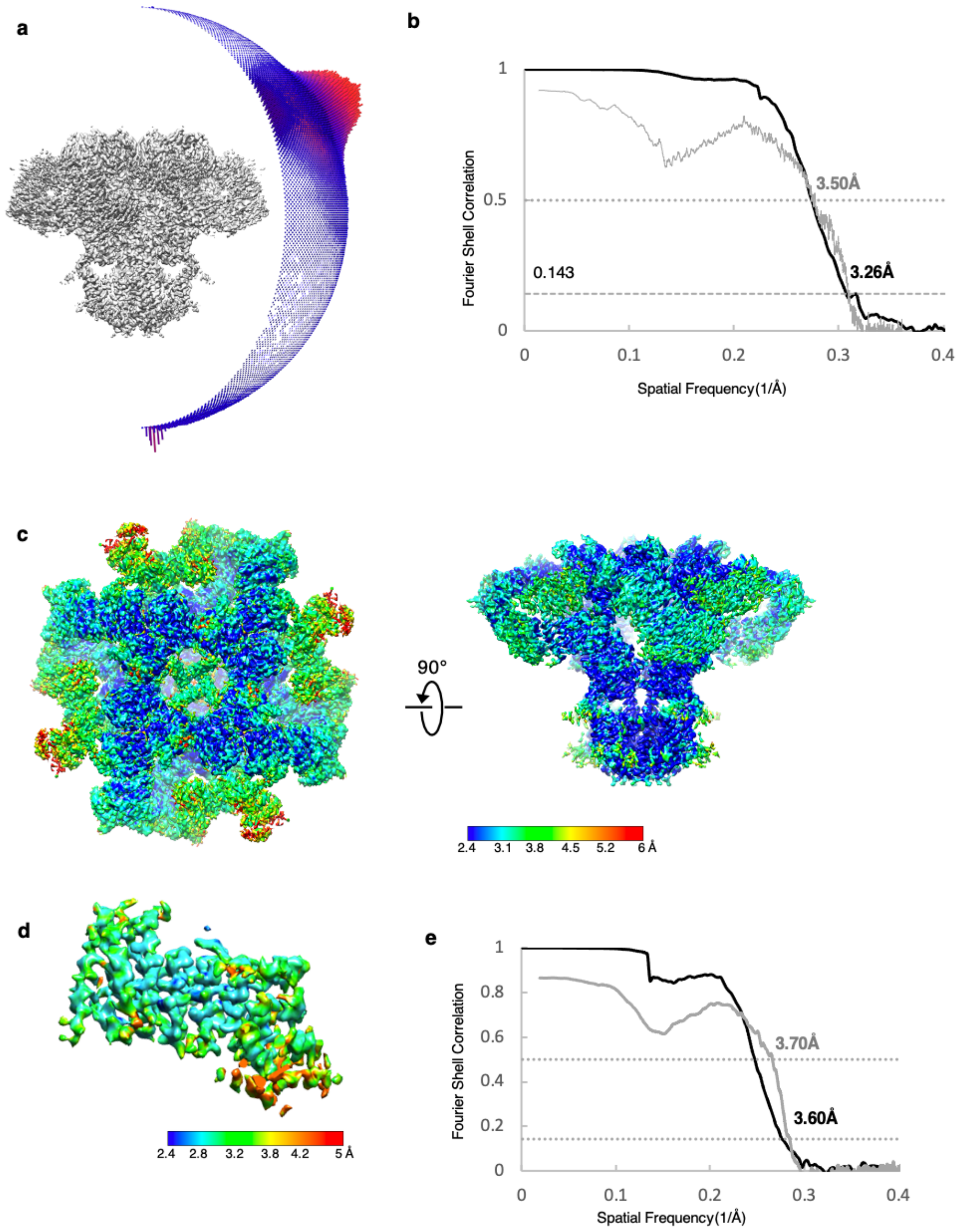

Supplementary Figure 2. Characteristics of cryo-EM reconstructions for Ca-IP$_3$R1. a, Euler angle distribution of particles contributing to the final reconstruction with larger red cylinders representing orientations comprising more particles. **b**, Resolution estimation of the cryo-EM density maps. Black – gold-standard Fourier shell correlation (FSC) curves, showing the overall nominal resolutions of 3.26 Å at 0.143 criterion; gray – Map-model FSC plot calculated by Phenix. **c**, Cryo-EM map colored based on local resolution. **d**, Focused 3D refined ARM2 map colored based on local resolution. **e**, Resolution estimation of the focused 3D refined ARM2 domain density map. Black – gold-standard Fourier shell correlation (FSC) curves, showing the overall nominal resolutions of 3.6 Å at 0.143 criterion; gray - Map-model FSC plot calculated by Phenix.

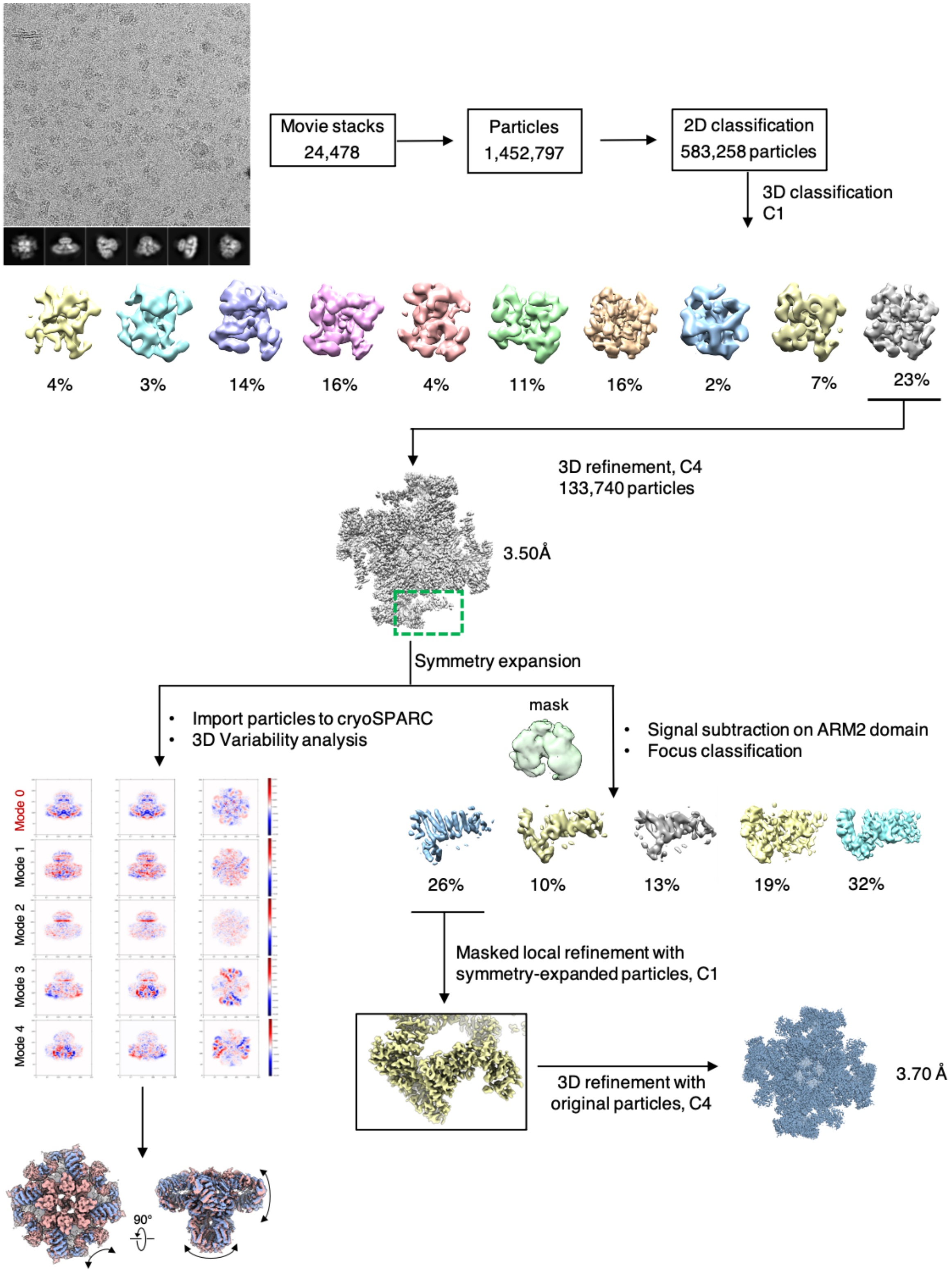

Supplementary Figure 3. Cryo-EM processing workflow for CIA-IP$_3$R1.

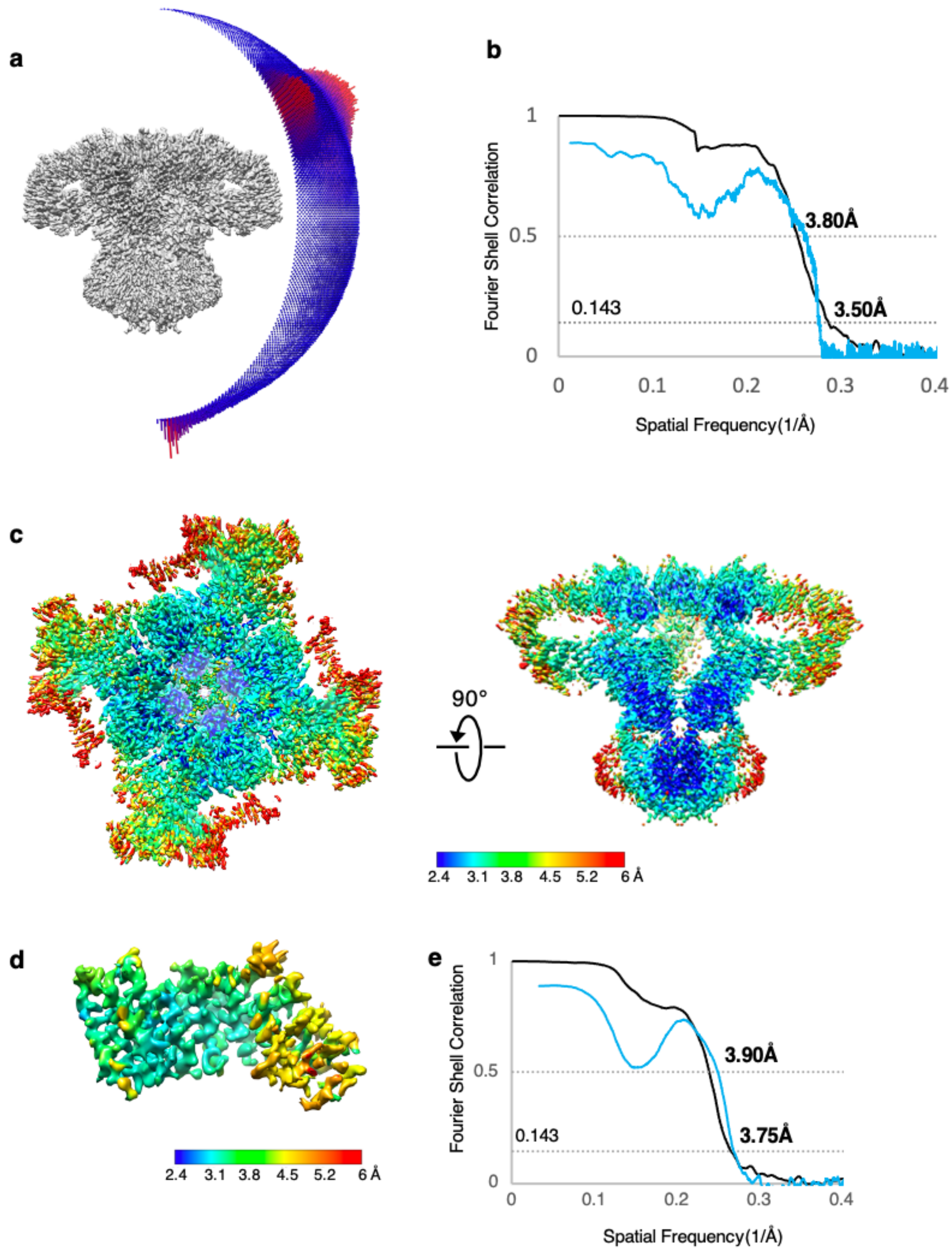

Supplementary Figure 4. Characteristics of cryo-EM reconstructions for CIA-IP$_3$R1. **a,** Euler angle distribution of particles contributing to the final reconstruction with larger red cylinders representing orientations comprising more particles. **b,** Resolution estimation of the cryo-EM density maps. Black – gold-standard Fourier shell correlation (FSC) curves, showing the overall nominal resolutions of 3.5 Å at 0.143 criterion; blue - Map-model FSC plot calculated by Phenix. **c,** Cryo-EM map colored based on local resolution. **d,** Focused 3D refined ARM2 map colored based on local resolution. **e**, Resolution estimation of the focused 3D refined ARM2 domain density map. Black – gold-standard Fourier shell correlation (FSC) curves, showing the overall nominal resolutions of 3.7 Å at 0.143 criterion; blue - Map-model FSC plot calculated by Phenix.

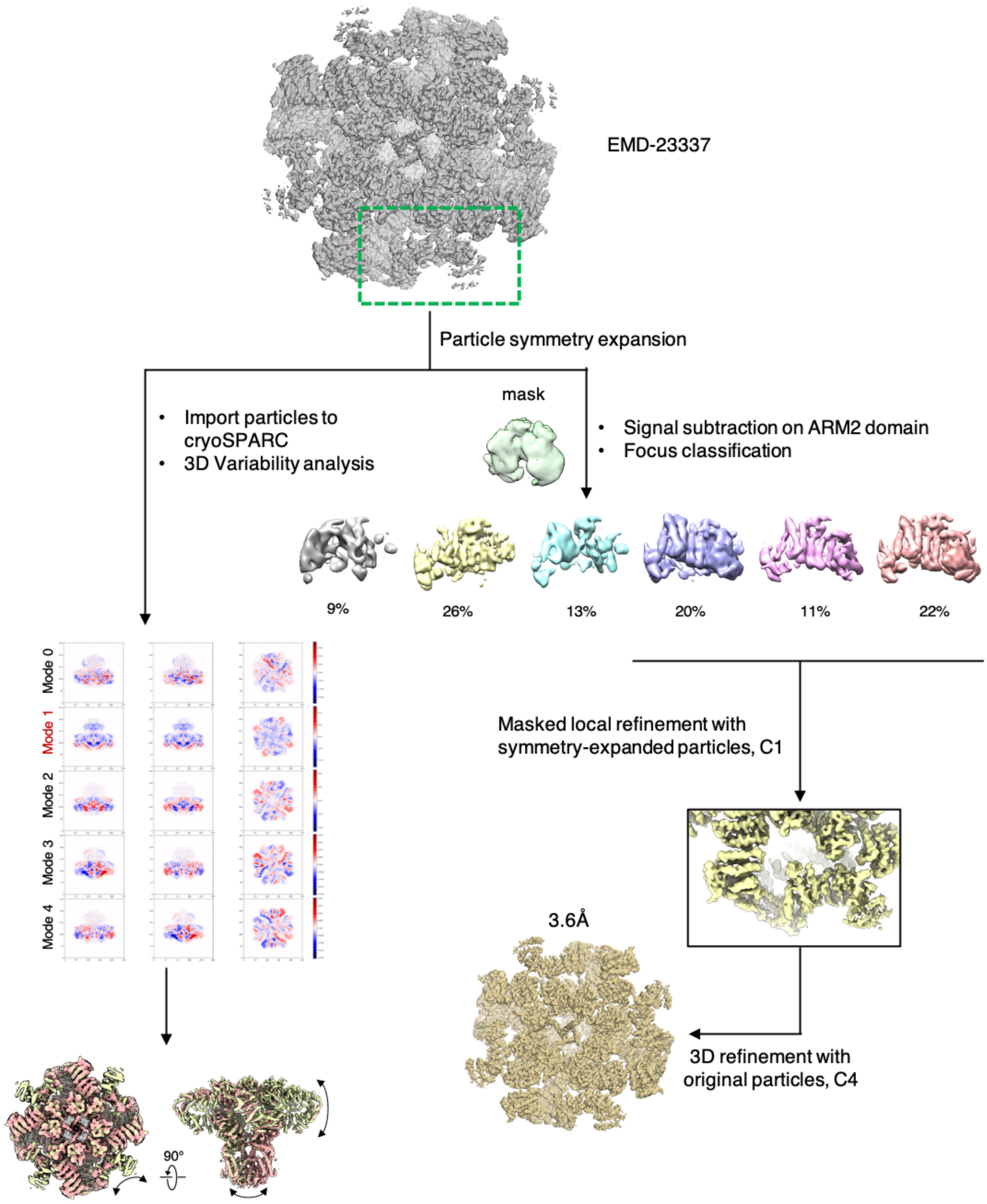

Supplementary Figure 5. 3D focused classification and local refinement on ARM2 domain for Apo-IP_3R1 in nanodisc used in Baker *et al.*[20]

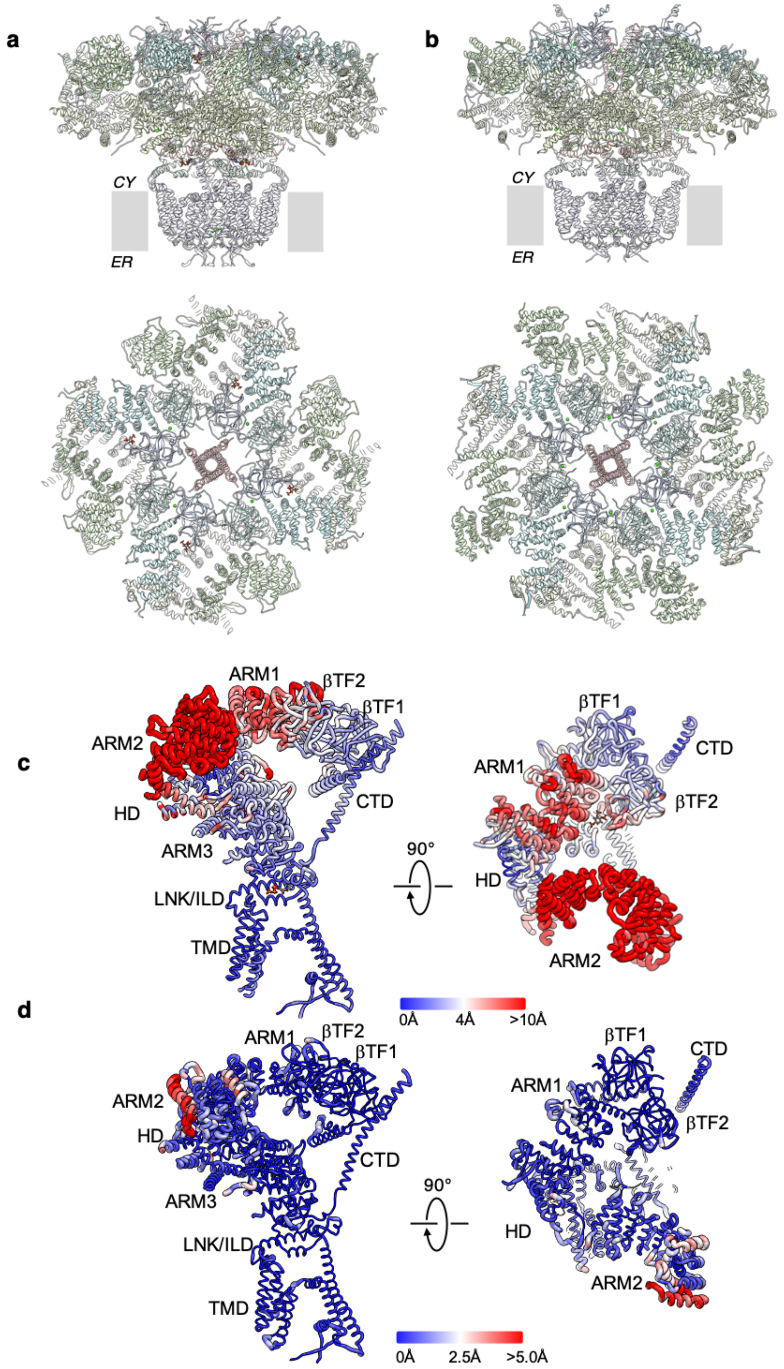

Supplementary Figure 6. Atomic models for the full-length tetrameric assembly of CIA-IP$_3$R1 (**a**) and Ca-IP$_3$R1 (**b**) based on the respective cryo-EM density maps. Top panels show side views along the membrane plane. Transmembrane boundaries are indicated in gray. Bottom panels show top-views from the cytosol along the four-fold axis. **c, d**, Cα RMS deviations were calculated between CIA-IP$_3$R1 and Ca-IP$_3$R1 (**c**) and Ca-IP$_3$R1 and apo-IP$_3$R1 (PDB ID: 7LHE) (**d**) resulting in 11 Å and 2 Å RMSD, respectively. Per-residue deviations are mapped onto one subunit and color-coded based on their deviation lowest RMSD (blue/thinnest) to highest RMSD (red/thickest).

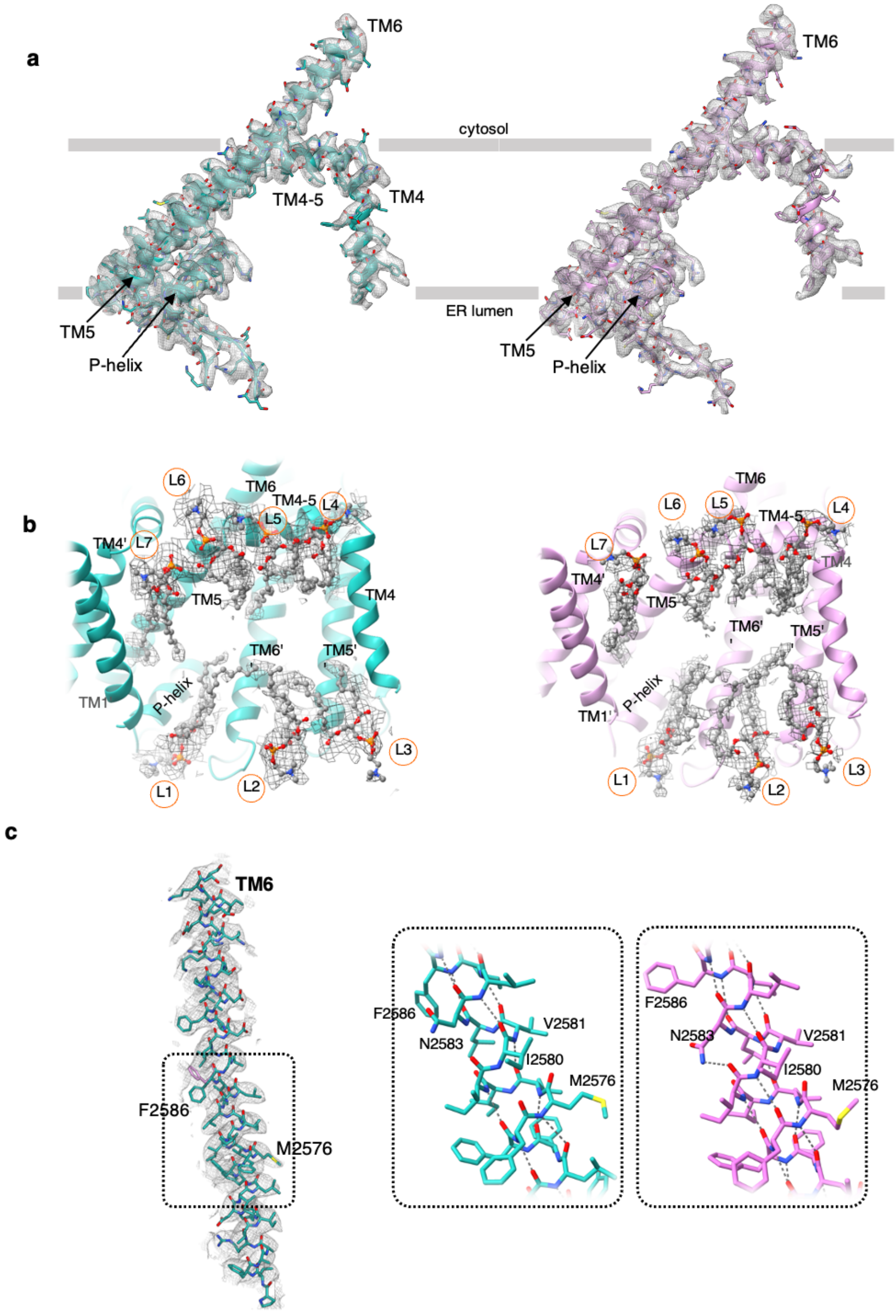

Supplementary Figure 7. Structural features in the IP_3R1 transmembrane domains. a, Densities (gray) for TMDs TM4 through TM6 from one subunit are overlaid with their atomic models CIA-IP_3R1 (blue-green), Ca-IP_3R1 (pink) and membrane boundaries indicated. **b,** Non-protein densities (gray mesh) within the transmembrane domains corresponding to lipids were identified in CIA-IP_3R1 (left) and Ca-IP_3R1 (right) are consistent with those described in Baker et al.[27]. The model of the TMDs is depicted as a ribbon and viewed parallel to the membrane plane. Lipids are represented as ball-and-stick models and labeled L1–L7. **c,** TM6 helix in CIA-IP_3R1 (blue-green) is overlaid with cryo-EM densities, colored gray (oxygen atoms - red; nitrogen atoms - blue; sulfur atoms - yellow); boxed region indicates the location for the π-helix in TM6. Zoomed-in views of the π-helix in CIA-IP_3R1 (blue-green) and Ca-IP_3R1 (pink) are shown in inserts.

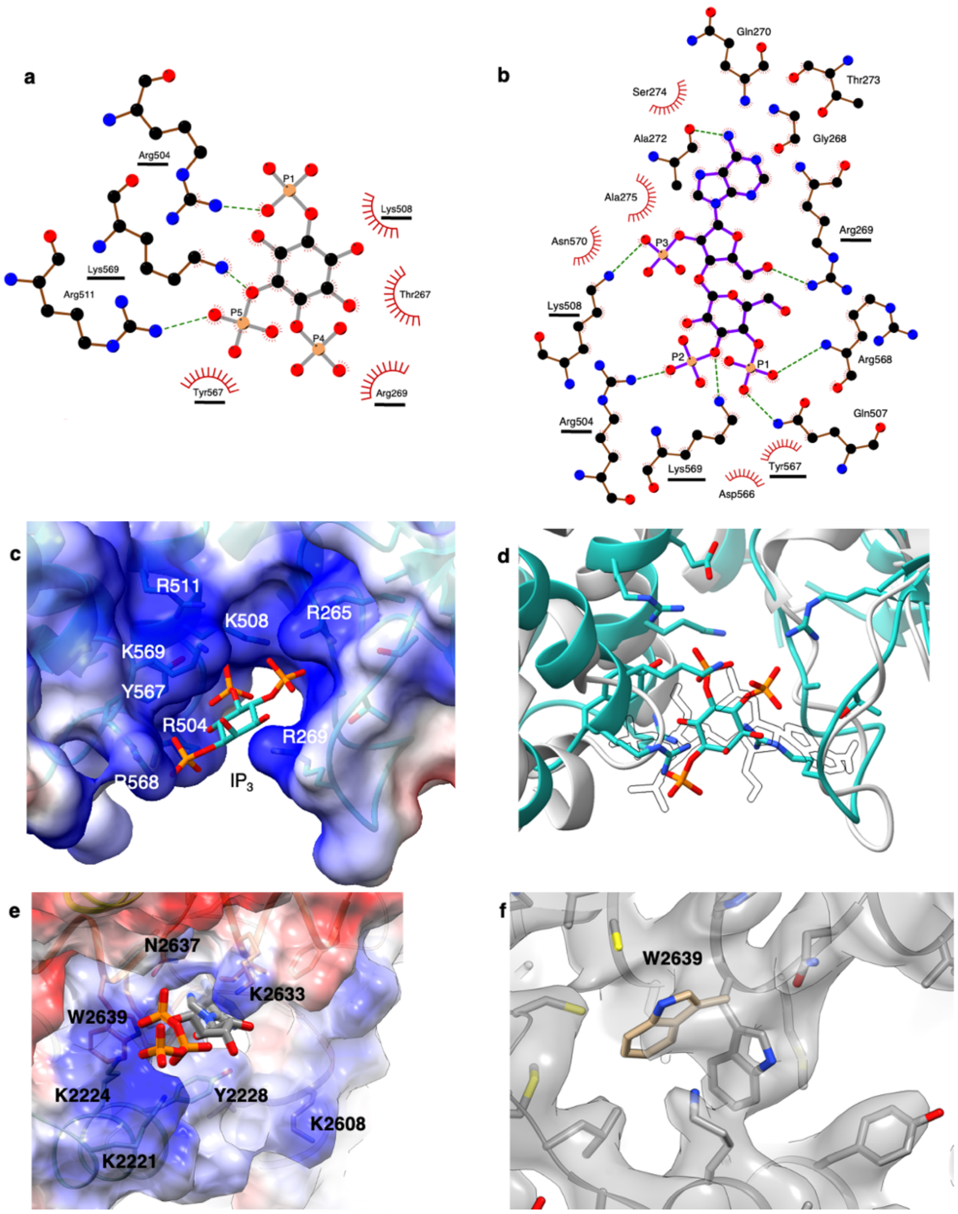

Supplementary Figure 8. Properties within the IP_3 and ATP ligand binding pockets. a, Schematic plot of the IP_3 molecule interacting with surrounding residues within the in CIA-IP_3R1 structure. **b,** Schematic plot of the Adenophostin A molecule interacting with surrounding residues in the ADA-IP_3R1 (PDB ID: 6MU1) structure. IP_3R1 residues that are consistent in coordinating ADA and IP_3 are underlined. Arcs represent hydrophobic interactions, green dashed lines are H-bonds as calculated in LigPlot+[80]. **c**, Coulombic electrostatic potential calculated and displayed on the surface representation of the IP_3 binding pocket in CIA-IP_3R1 with the pocket lining residues labeled. Blue surfaces indicate positive charges and red surfaces indicate negative charges. **d,** Structural alignment and overlay of the IP_3 binding pocket in CIA-IP_3R1 (blue-green) and ADA-IP_3R1 (PDB ID: 6MU1, white). **e**, Coulombic electrostatic potential calculated and displayed on the surface representation of the ATP binding pocket in CIA-IP_3R1 with the pocket lining residues labeled. **f**, Zoomed-in view of the ATP binding pocket in apo-IP_3R1 (EMDB: 23337) overlaid with its molecular model (PBD ID: 7LHE) and focused on the side chain conformers for W2639.

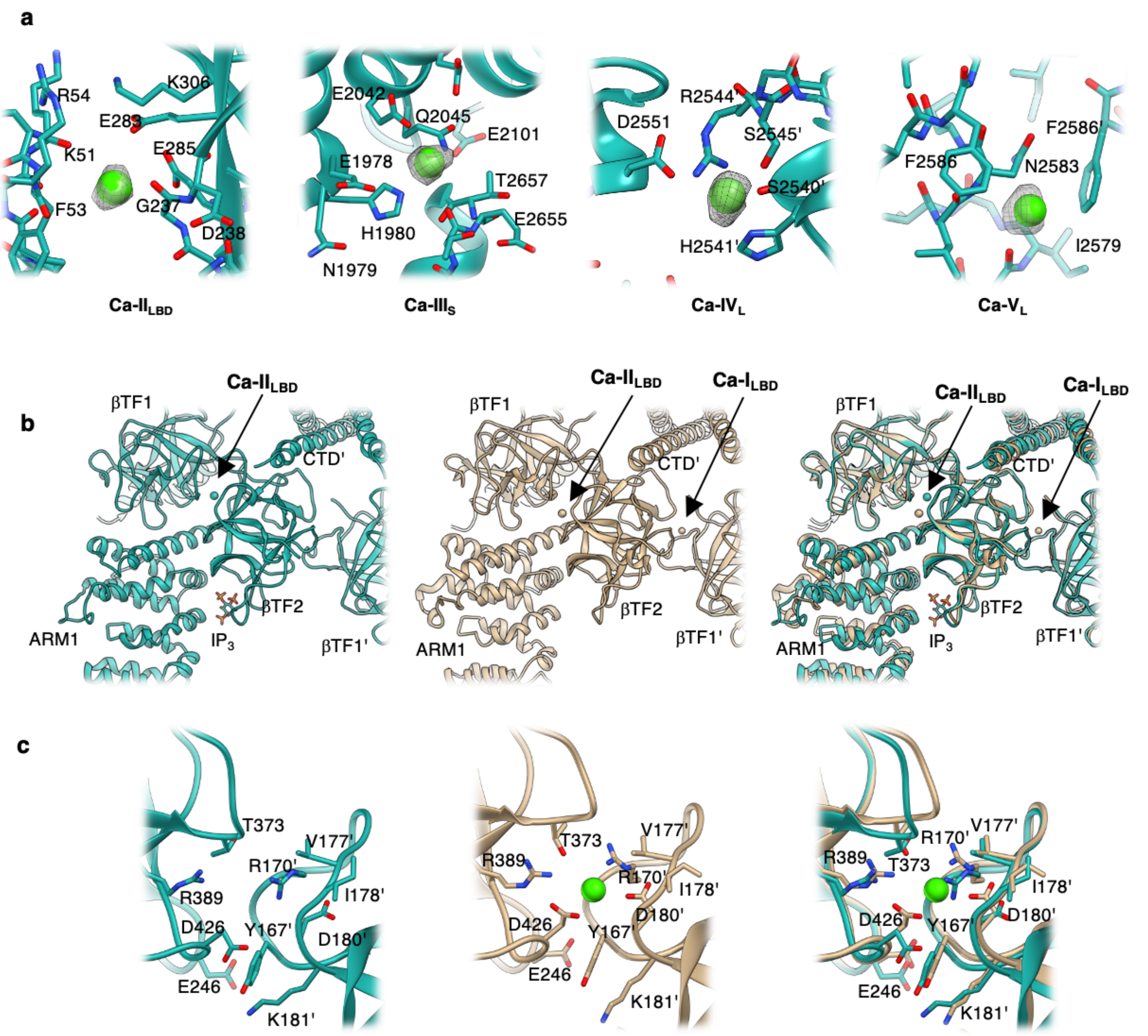

Supplementary Figure 9. Ca^{2+} binding sites identified in CIA-IP$_3$R1. a, Ca^{2+} binding sites localized in the ligand binding domains (Ca-II$_{LBD}$), Ca^{2+} sensor/ARM3 domain (Ca-III$_S$), and luminal vestibule of the TMD (Ca-IV$_L$ and Ca-V$_L$). Ca^{2+} ions are shown as green spheres and overlaid with corresponding densities displayed at 2-5 σ cutoff values. Residues within 5 Å of the Ca^{2+} ions are displayed in a stick representation and labeled. **b**, Comparison of Ca^{2+}-binding sites identified in ligand binding domains in CIA-IP$_3$R1 (blue-green) and Ca-IP$_3$R1 (tan). **c**, Zoom-in views of Ca-I$_{LBD}$ binding sites in CIA-IP$_3$R1 (blue-green) and Ca-IP$_3$R1 (tan).
