# Supplementary material for: Conformational motions and ligand-binding underlying gating and regulation in IP3R channel
Source: Nat Commun. 2022 Nov 14;13:6942. doi: 10.1038/s41467-022-34574-1 (PMC9663519; doi:10.1038/s41467-022-34574-1)
Supplement: Supplementary file 3 — Description of Additional Supplementary Files [file 41467_2022_34574_MOESM3_ESM.pdf]

Description of Additional Supplementary Files

File Name: Supplementary Movie 1
Description: **Three-dimensional variability analysis shows motions of CIA-IP$_3$R1 and Ca-**IP$_3$R1 (the structure is viewed along the membrane plane).

File Name: Supplementary Movie 2
Description: **Three-dimensional variability analysis shows motions in CIA-IP$_3$R1** viewed along the channel four-fold axis from cytosol.

File Name: Supplementary Movie 3
Description: **Three-dimensional variability analysis shows motions of CIA-IP$_3$R1** in nexus region viewed from cytosol (left) and lumen (right).
