# Supplementary material for: Conformational motions and ligand-binding underlying gating and regulation in IP3R channel
Source: Nat Commun. 2022 Nov 14;13:6942. doi: 10.1038/s41467-022-34574-1 (PMC9663519; doi:10.1038/s41467-022-34574-1)
Supplement: Supplementary file 7 — Reporting Summary [file 41467_2022_34574_MOESM7_ESM.pdf]

nature portfolio

Corresponding author(s): Irina I. Serysheva
David I. Yule
NCOMMS-22-25452

Last updated by author(s): 2022/10/24

Reporting Summary

Nature Portfolio wishes to improve the reproducibility of the work that we publish. This form provides structure for consistency and transparency in reporting. For further information on Nature Portfolio policies, see our Editorial Policies and the Editorial Policy Checklist.

Statistics

For all statistical analyses, confirm that the following items are present in the figure legend, table legend, main text, or Methods section.

n/a	Confirmed	
☐	☒	The exact sample size (*n*) for each experimental group/condition, given as a discrete number and unit of measurement
☐	☒	A statement on whether measurements were taken from distinct samples or whether the same sample was measured repeatedly
☐	☒	The statistical test(s) used AND whether they are one- or two-sided<br>*Only common tests should be described solely by name; describe more complex techniques in the Methods section.*
☒	☐	A description of all covariates tested
☒	☐	A description of any assumptions or corrections, such as tests of normality and adjustment for multiple comparisons
☐	☒	A full description of the statistical parameters including central tendency (e.g. means) or other basic estimates (e.g. regression coefficient) AND variation (e.g. standard deviation) or associated estimates of uncertainty (e.g. confidence intervals)
☐	☒	For null hypothesis testing, the test statistic (e.g. *F*, *t*, *r*) with confidence intervals, effect sizes, degrees of freedom and *P* value noted<br>*Give P values as exact values whenever suitable.*
☒	☐	For Bayesian analysis, information on the choice of priors and Markov chain Monte Carlo settings
☒	☐	For hierarchical and complex designs, identification of the appropriate level for tests and full reporting of outcomes
☒	☐	Estimates of effect sizes (e.g. Cohen's *d*, Pearson's *r*), indicating how they were calculated

Our web collection on statistics for biologists contains articles on many of the points above.

Software and code

Policy information about availability of computer code

Data collection	EPU (Thermo Fisher Inc.) 2.10.0.5, TILL Photonics /TILLvisION Ver 4.5.66
Data analysis	cryoSPARC v3.2, RELION3.0.8, EMAN2.31 and 2.91, Coot 0.9.5, MotionCor2_1.2.2, Gctf 1.06, UCSF Chimera 1.16 and ChimeraX 1.2, Phenix 1.19.1-4122, pw_ligands.py v1.0, HOLE 2.2.005, Ligplot+ v2.2, PDBePisa v1.52, Alphafold2_Advanced (22/1/28), Microsoft Excel 16.66.1, GraphPad Prism 8.0, VMD 1.9.4, Studio Lite Ver 5.2

For manuscripts utilizing custom algorithms or software that are central to the research but not yet described in published literature, software must be made available to editors and reviewers. We strongly encourage code deposition in a community repository (e.g. GitHub). See the Nature Portfolio guidelines for submitting code & software for further information.

Data

Policy information about availability of data

All manuscripts must include a data availability statement. This statement should provide the following information, where applicable:

- Accession codes, unique identifiers, or web links for publicly available datasets
- A description of any restrictions on data availability
- For clinical datasets or third party data, please ensure that the statement adheres to our policy

Data supporting the findings of this manuscript are available from the corresponding author upon reasonable request. Coordinates and corresponding EM maps of CIA-IP3R1 (PDB: 8EAR [https://doi.org/10.2210/pdb8EAR/pdb]; EMDB: EMD-27983 [https://www.ebi.ac.uk/emdb/EMD-27983]) and Ca-IP3R1 (PDB: 8EAQ [https://doi.org/10.2210/pdb8EAQ/pdb]; EMDB: EMD-27982 [https://www.ebi.ac.uk/emdb/EMD-27982]) have been deposited in the Protein Data Bank (http://

www.rcsb.org) and the Electron Microscopy Data Bank (https://www.ebi.ac.uk/pdbe/emdb/), respectively. Data from our previously resolved cryo-EM map of apo-IP3R1 (Electron Microscopy Data Bank (EMDB): EMD-23337 [https://www.ebi.ac.uk/emdb/EMD-23337] and coordinates (PDB ID: 7LHE; [http://10.2210/pdb7lhe/pdb]) were used in this study. A source data file is provided for Figure 6.

Human research participants

Policy information about studies involving human research participants and Sex and Gender in Research.

Reporting on sex and gender	N/A
Population characteristics	N/A
Recruitment	N/A
Ethics oversight	N/A

Note that full information on the approval of the study protocol must also be provided in the manuscript.

Field-specific reporting

Please select the one below that is the best fit for your research. If you are not sure, read the appropriate sections before making your selection.

☒ Life sciences ☐ Behavioural & social sciences ☐ Ecological, evolutionary & environmental sciences

For a reference copy of the document with all sections, see nature.com/documents/nr-reporting-summary-flat.pdf

Life sciences study design

All studies must disclose on these points even when the disclosure is negative.

Sample size	For Ca-IP3R1 data set 1,955,320 particles were selected from 25,125 movie stacks. For CIA-IP3R1 data set 1,452,797 particles were selected from 24,475 movie stacks. No statistical methods were used to determine EM data sample size.
Data exclusions	Iterative 2D and 3D classifications were used to eliminate particles.
Replication	Reproducibility of the final 3D maps was assessed using the gold-standard Fourier shell correlation 0.143 criterion
Randomization	Randomization is implemented in image processing. Individual particle data sets were randomly split into odd and even halves and refined independently.
Blinding	Data was not blinded and is not applicable to cryoEM studies.

Reporting for specific materials, systems and methods

We require information from authors about some types of materials, experimental systems and methods used in many studies. Here, indicate whether each material, system or method listed is relevant to your study. If you are not sure if a list item applies to your research, read the appropriate section before selecting a response.

Materials & experimental systems

n/a	Involved in the study	
☐	☒	Antibodies
☐	☒	Eukaryotic cell lines
☒	☐	Palaeontology and archaeology
☒	☐	Animals and other organisms
☒	☐	Clinical data
☒	☐	Dual use research of concern

Methods

n/a	Involved in the study	
☒	☐	ChIP-seq
☒	☐	Flow cytometry
☒	☐	MRI-based neuroimaging

Antibodies

Antibodies used	Mouse monoclonal antibody to the T433 peptide derived antibody of the C-term of IP3R1 were produced commercially and 10-20 mg IgG utilized for immunoaffinity purification. Rabbit polyclonal antibody against the C-terminal 19aa of IP3R1 (custom generated by Antibody Research Corporation) at a 1:1000 dilution, GAPDH (#AM4300, Invitrogen) at a 1:75,000 dilution, and the appropriate Dylight™ 800CW secondary antibodies at a 1:10,000 dilution (SA535571 and SA535521; Invitrogen).
Validation	IP3R1 antibodies were validated based on western blot analysis of rat cerebella microsomal membranes. No additional validations were performed.

Eukaryotic cell lines

Policy information about cell lines and Sex and Gender in Research

Cell line source(s)	HEK-293s were originally obtained from ATCC. HEK-3KO cells were created by CRISPR-Cas9 gene editing to delete all alleles of IP3R. The mutant IP3R were stably expressed on this null background. Endo. hR1 expressing HEK cells were generated by gene editing and deletion of IP3R2 and IP3R3 alleles. (Details in Alzayady et al., Science Signaling 9. 422. 2016)
Authentication	Deletion of all IP3R alleles in the HEK-3KO was confirmed by western blotting and genotyping confirmed the successful gene editing. No further authentication was carried out.
Mycoplasma contamination	tested and negative
Commonly misidentified lines (See ICLAC register)	NO
