# Supplementary figures and images for: Conformational motions and ligand-binding underlying gating and regulation in IP3R channel
Source: Nat Commun. 2022 Nov 14;13:6942. doi: 10.1038/s41467-022-34574-1 (PMC9663519; doi:10.1038/s41467-022-34574-1)

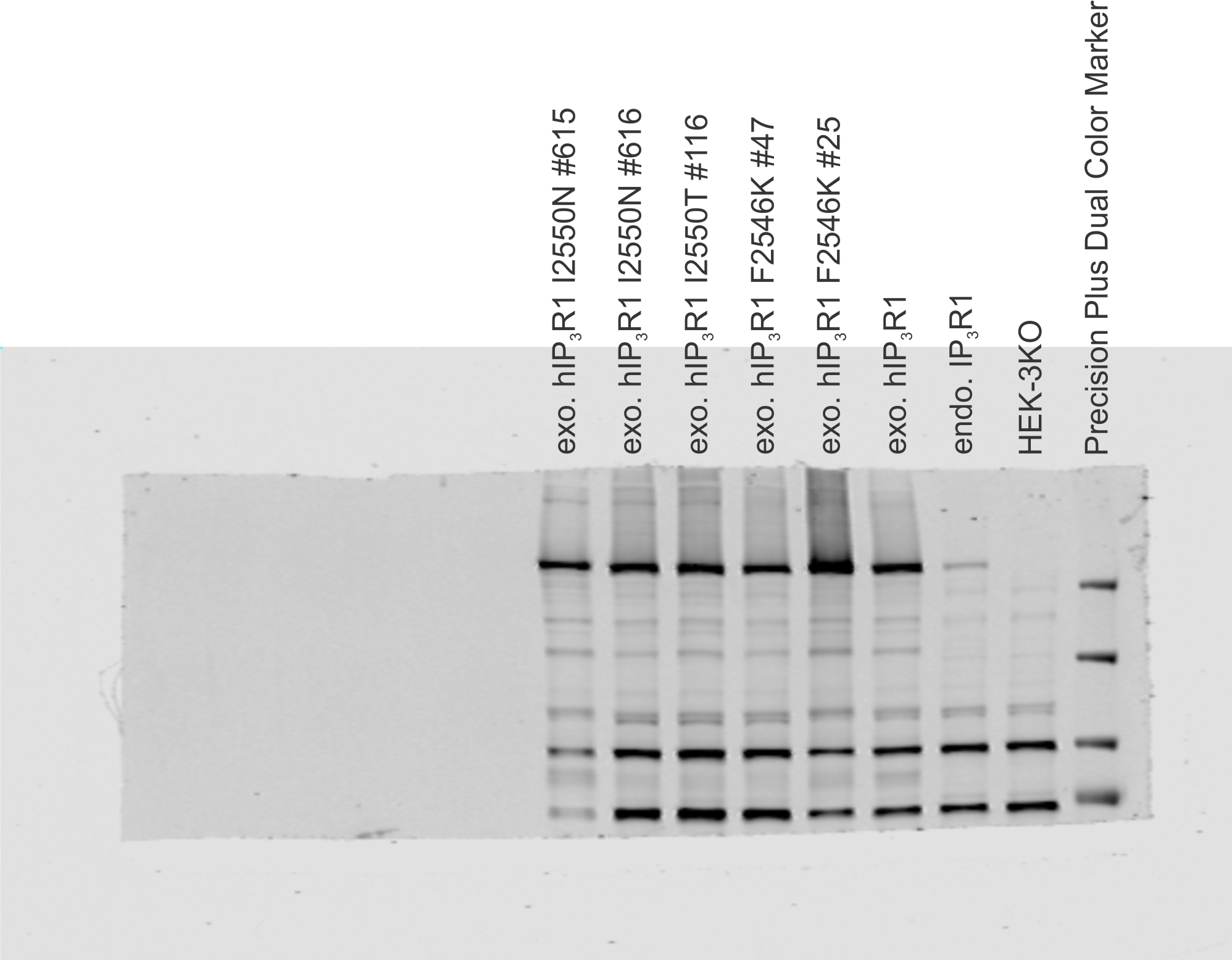

Supplement: Supplementary file 8 — Source Data [file 41467_2022_34574_MOESM8_ESM.zip › Figure 6a Blots - labeled/Figure 6a - Blot 1 - Anti-IP3R1 - Labeled.tif]

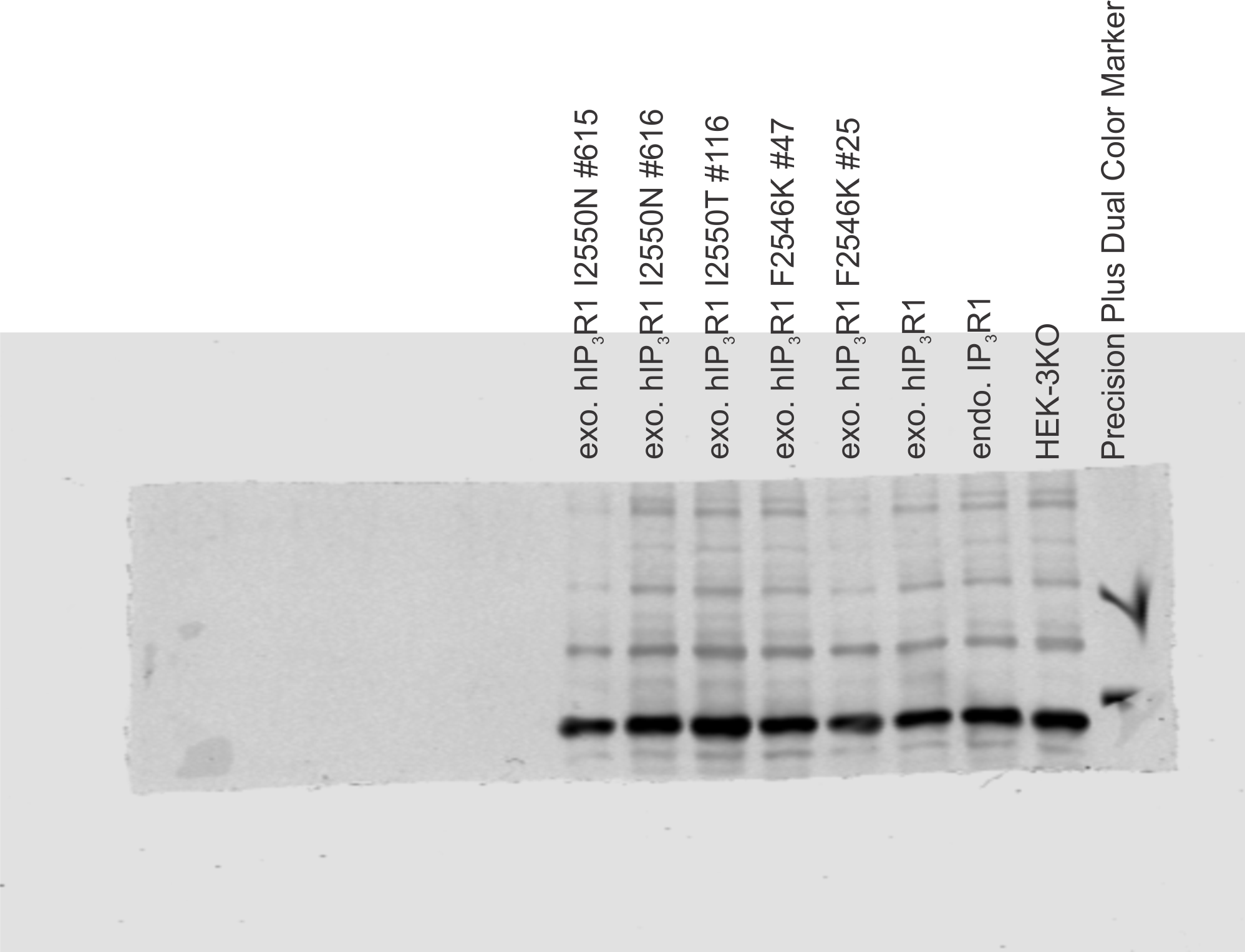

Supplement: Supplementary file 8 — Source Data [file 41467_2022_34574_MOESM8_ESM.zip › Figure 6a Blots - labeled/Figure 6a - Blot 1 - Anti-GAPDH - Labeled.tif]

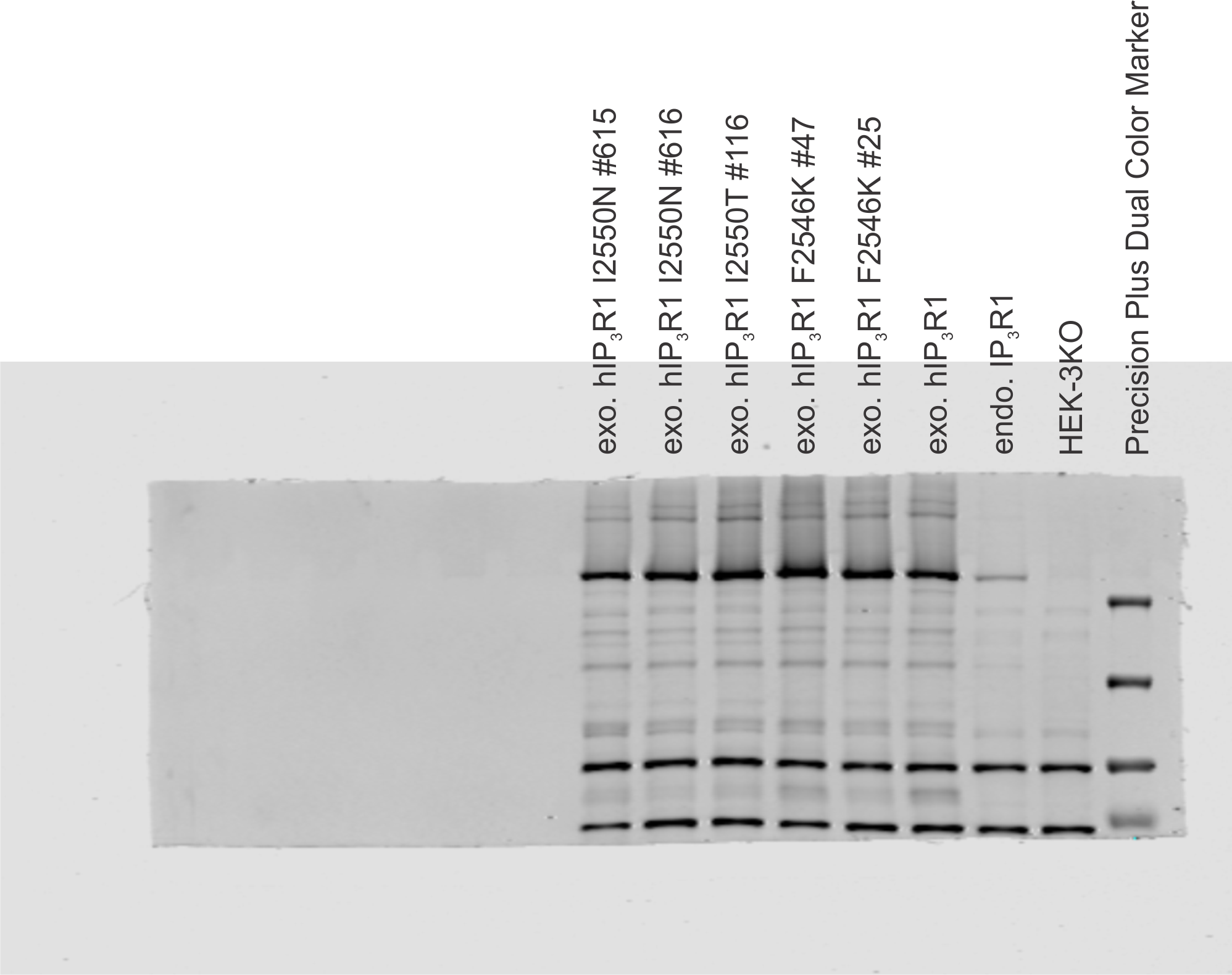

Supplement: Supplementary file 8 — Source Data [file 41467_2022_34574_MOESM8_ESM.zip › Figure 6a Blots - labeled/Figure 6a - Blot 3 - Anti-IP3R1 - Labeled.tif]

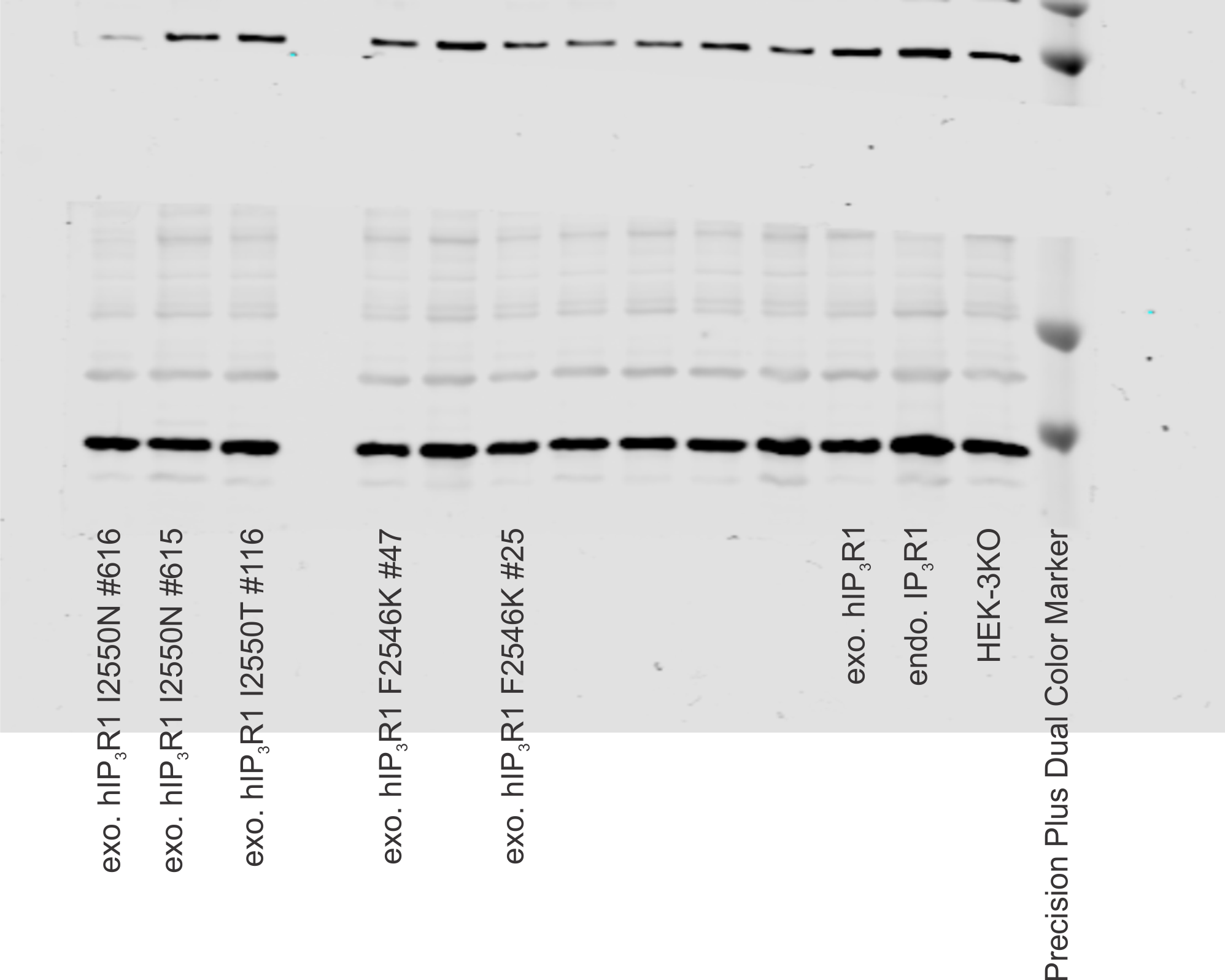

Supplement: Supplementary file 8 — Source Data [file 41467_2022_34574_MOESM8_ESM.zip › Figure 6a Blots - labeled/Figure 6a - Blot 4 - Anti-GAPDH - Labeled.tif]

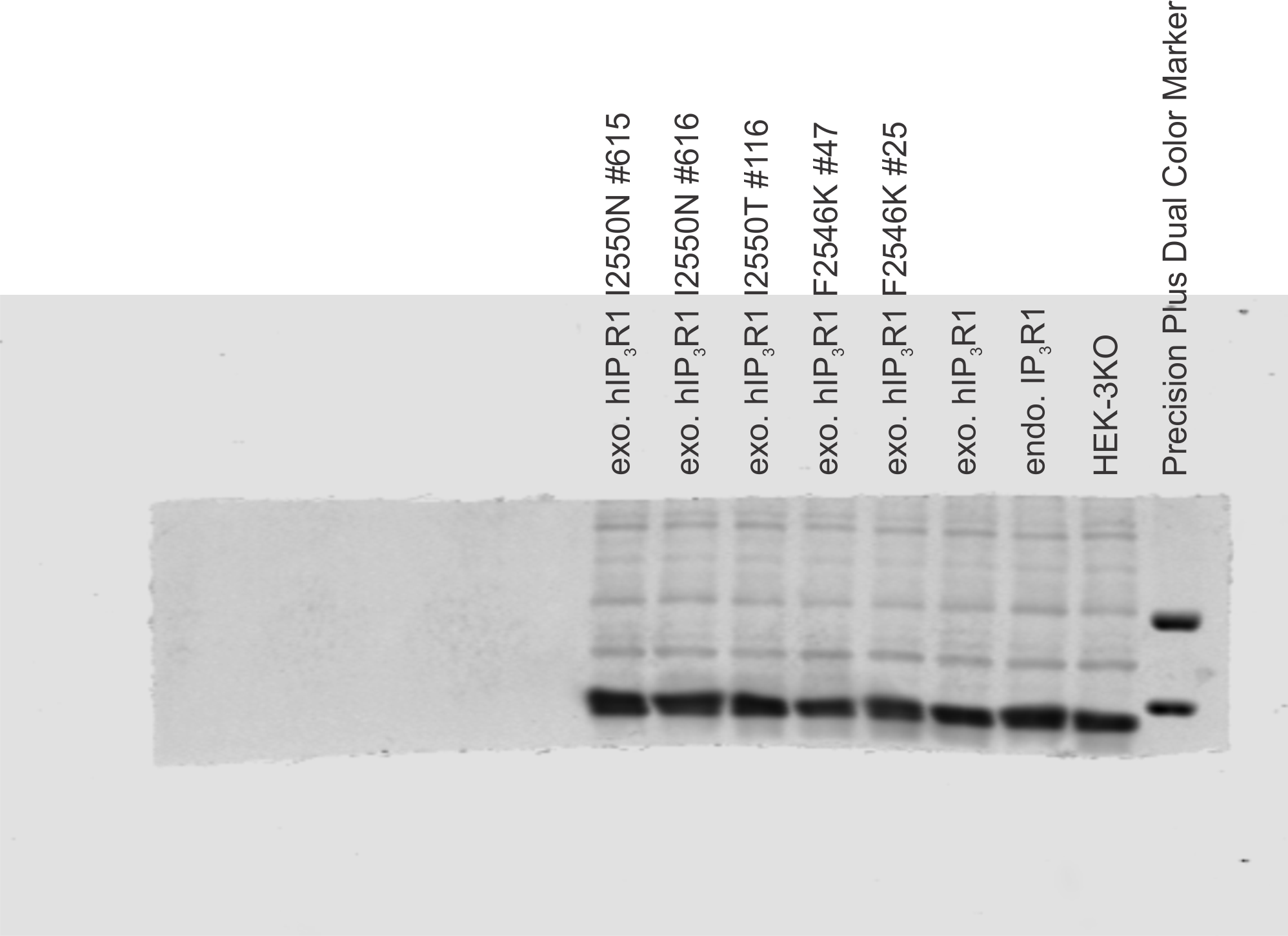

Supplement: Supplementary file 8 — Source Data [file 41467_2022_34574_MOESM8_ESM.zip › Figure 6a Blots - labeled/Figure 6a - Blot 3 - Anti-GAPDH - Labeled.tif]

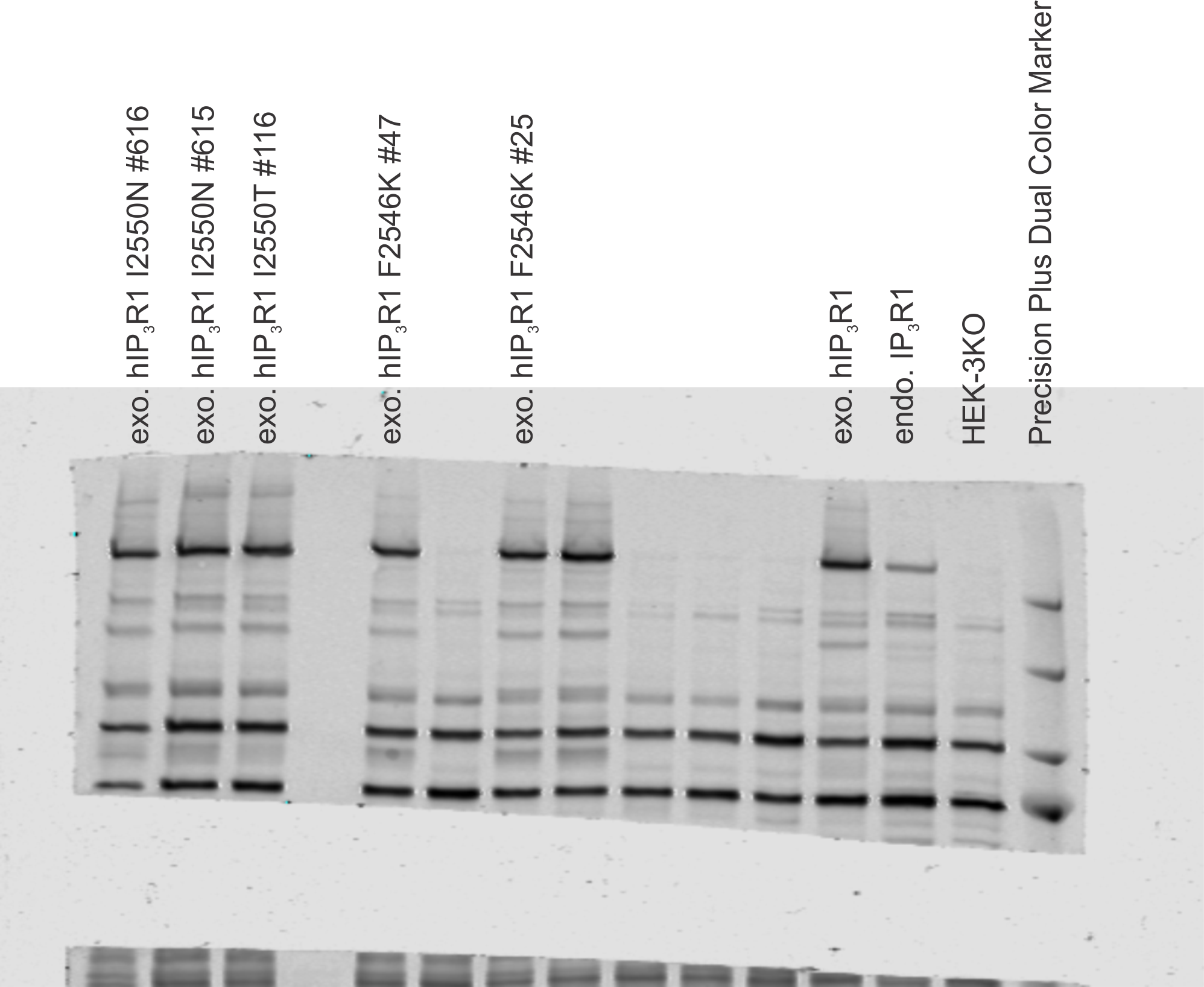

Supplement: Supplementary file 8 — Source Data [file 41467_2022_34574_MOESM8_ESM.zip › Figure 6a Blots - labeled/Figure 6a - Blot 4 - Anti-IP3R1 - labeled.tif]

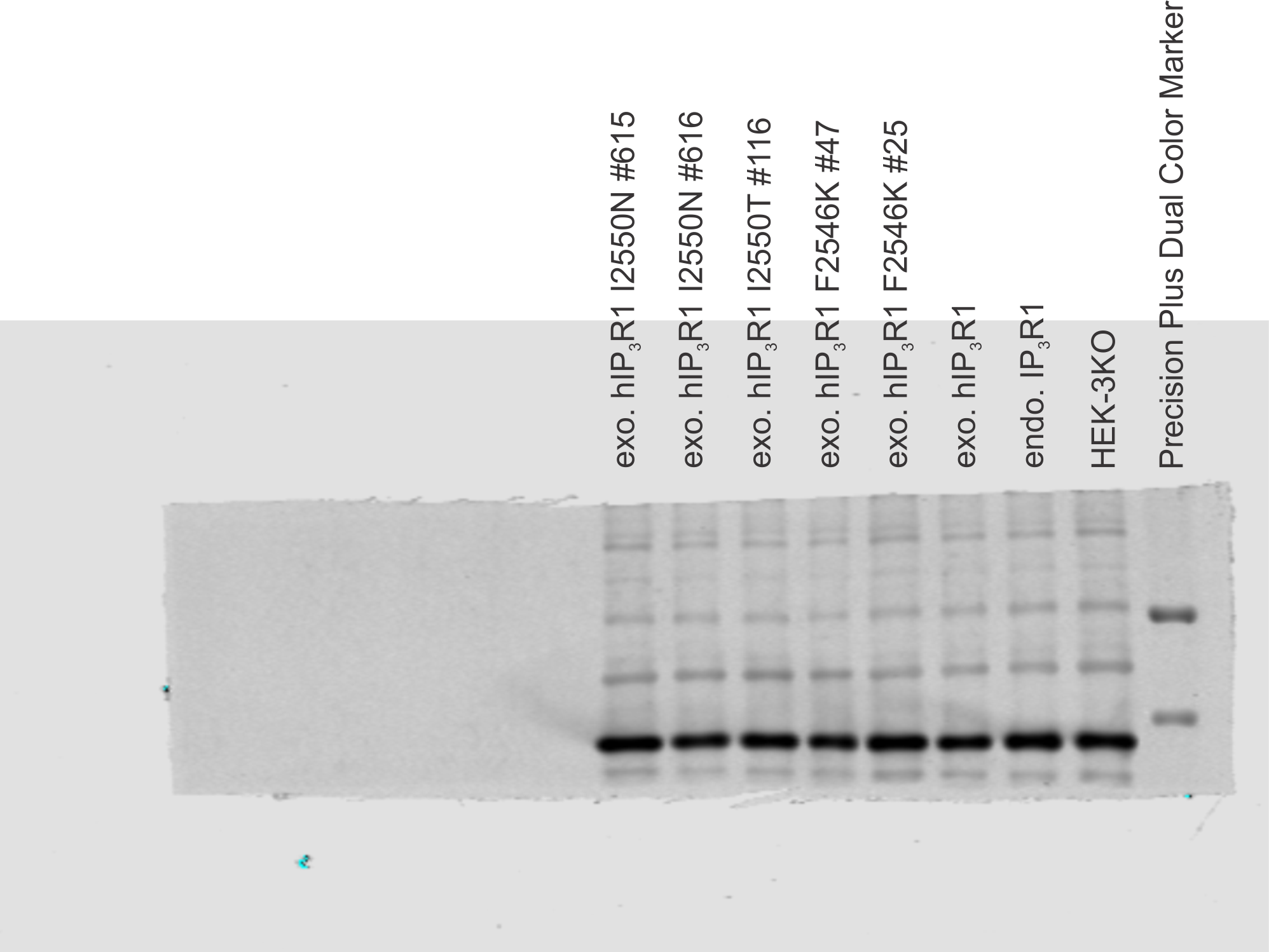

Supplement: Supplementary file 8 — Source Data [file 41467_2022_34574_MOESM8_ESM.zip › Figure 6a Blots - labeled/Figure 6a - Blot 2 - Anti-GAPDH - Labeled.tif]

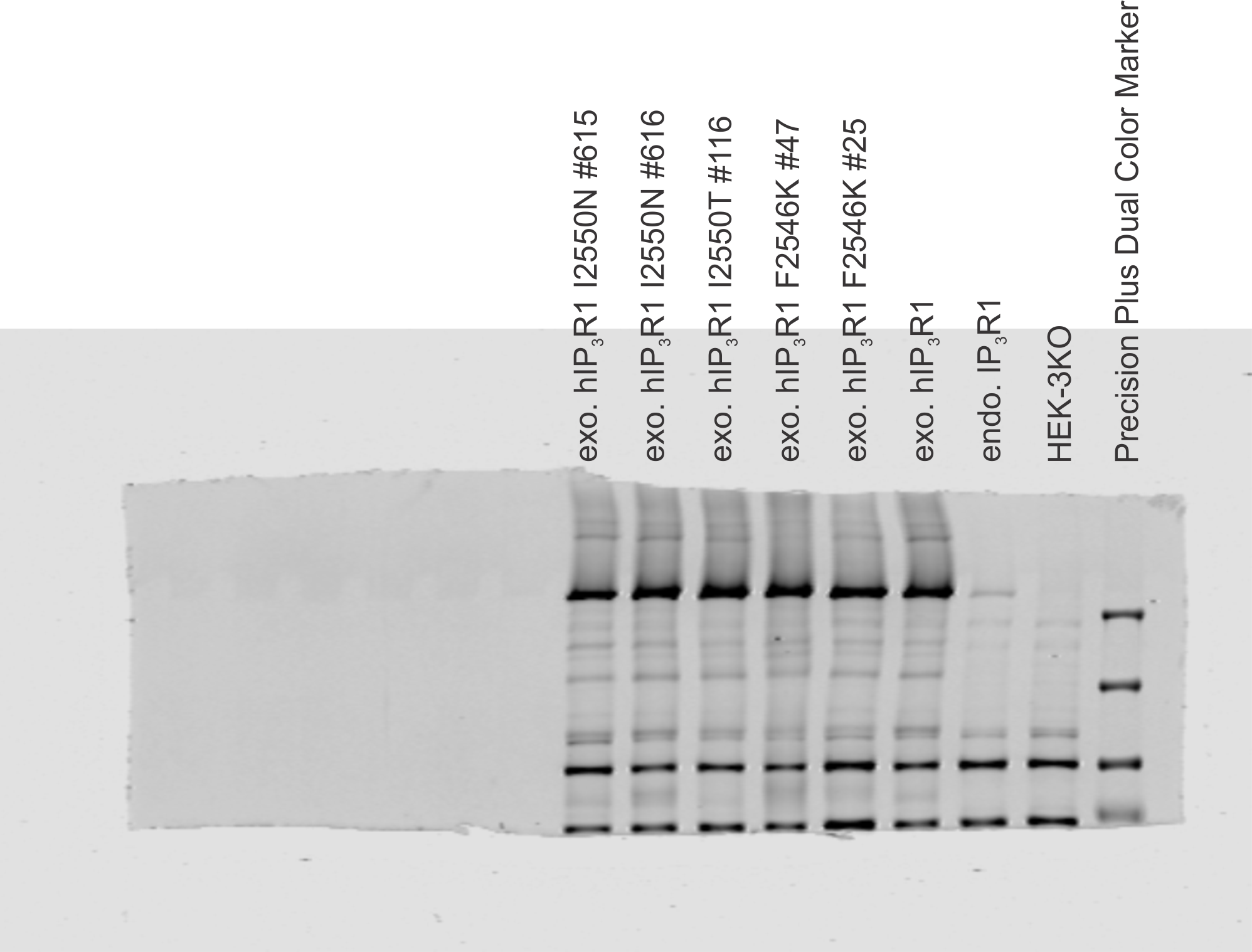

Supplement: Supplementary file 8 — Source Data [file 41467_2022_34574_MOESM8_ESM.zip › Figure 6a Blots - labeled/Figure 6a - Blot 2 - Anti-IP3R1 - Labeled.tif]
